# A comprehensive lemongrass (*Cymbopogon citratus*) leaf dataset for agricultural research and disease prevention

**DOI:** 10.1016/j.dib.2024.110104

**Published:** 2024-01-30

**Authors:** Kailas Patil, Yogesh Suryawanshi, Alimurtuza Patrawala, Prawit Chumchu

**Affiliations:** a Vishwakarma University, Pune, India; b Kasetsart University, Sriracha, Thailand

**Keywords:** *Cymbopogon citratus*, Disease prevention, Lemongrass leaves, Leaf assessment, Plant health analysis

## Abstract

This article introduces a dataset of 10,042 Lemongrass (*Cymbopogon citratus*) leaf images, captured with high quality camera of a mobile phone in real-world conditions. The dataset classifies leaves as “Dried,” “Healthy,” or “Unhealthy,” making it useful for machine learning, agriculture research, and plant health analysis. We collected the plant leaves from the Vishwakarma University Pune herbal garden and the captured the images in diverse backgrounds, angles, and lighting conditions. The images underwent pre-processing, involving batch image resizing through FastStone Photo Resizer and subsequent operations for compatibility with pre-trained models using the ‘preprocess_input’ function in the Keras library. The significance of the Lemongrass Leaves Dataset was demonstrated through experiments using well-known pre-trained models, such as InceptionV3, Xception, and MobileNetV2, showcasing its potential to enhance machine learning model accuracy in Lemongrass leaf identification and disease detection. Our goal is to aid researchers, farmers, and enthusiasts in improving Lemongrass cultivation and disease prevention. Researchers can use this dataset to train machine learning models for leaf condition classification, while farmers can monitor their crop's health. Its authenticity and size make it valuable for projects enhancing Lemongrass cultivation, boosting crop yield, and preventing diseases. This dataset is a significant step toward sustainable agriculture and plant health management.

Specifications Table

**Table:** 

Subject	Applied Machine Learning, Agriculture
Specific subject area	Agronomy & Crop Science
Data format	Raw
Type of data	Image
Data collection	The dataset, consisting of 10,042 images of lemon grass leaves, was meticulously collected at Vishwakarma University. These leaves were sourced from local gardens and our own homegrown plants, representing three distinct conditions: dried, healthy, and unhealthy. To ensure a diverse range of images, deliberate efforts were made to incorporate variations in lighting, background settings, leaf positioning, and also captured images from different angles during the image capture process. Each individual leaf was meticulously photographed under controlled conditions, considering multiple lighting sources, both natural and artificial, and a diverse array of backgrounds to represent its condition accurately from various perspectives. The images that were taken were saved in JPG format and resized to a resolution of 1024 × 768 pixels.
Data source location	Vishwakarma University, Kondhwa Budruk, Maharashtra, Pune, India Latitude: 18.4605° N Longitude: 73.8837° E
Data accessibility	Repository name: Lemongrass Leaf Image Dataset: Mobile-Photographed Image Compilation Data identification number: 10.17632/9tnbjsj6kn.1 Direct URL to data: https://data.mendeley.com/datasets/9tnbjsj6kn/1

## 1 Value of the Data

• This dataset helps answer fundamental questions related to leaf quality assessment. It addresses the importance of data on Lemongrass leaves, the significance of studying plant diseases, potential technological advancements, and the broader areas where this data can be applied.
• Researchers and data scientists can utilize this dataset to develop and train machine learning algorithms for the classification of Lemongrass leaves into “Dried,” “Healthy,” and “Unhealthy” categories. This can pave the way for automated, accurate plant health assessment.
• The dataset serves as a valuable resource for early disease prevention in Lemongrass cultivation. Timely detection of unhealthy leaves can lead to more effective disease management strategies, reducing crop losses and the need for chemical interventions.
• Scientists and researchers can leverage this dataset to conduct in-depth studies on Lemongrass health, disease detection, and crop management.
• The dataset can be applied in developing a machine learning algorithm that accurately classifies Lemongrass leaves, aiding in automated plant health assessment. This technology enables precise monitoring and timely intervention, showcasing its potential for widespread agricultural impact.
• Applications can be developed using this dataset for crop monitoring that can help farmers for real-time monitoring of Lemongrass crops, helping them detect and address plant health issues promptly, thereby potentially increasing crop yield and quality.

This Lemongrass leaf dataset is a valuable resource with applications in research, agriculture, technology development, and disease prevention. It not only addresses essential questions but also provides practical tools for improving crop management and plant health assessment, ultimately contributing to more sustainable and efficient agricultural practices.

## 2 Data Description

The “*A Comprehensive Lemongrass Leaf Dataset for Agricultural Research and Disease Prevention*” serves as an invaluable resource for advancing agricultural research and disease prevention efforts [1]. This dataset is designed to enhance our understanding of lemongrass leaves and their conditions, particularly in the context of agricultural applications and disease prevention.

Containing a rich collection of high-quality lemongrass leaf images, meticulously gathered from Vishwakarma University, this dataset aims to support the development, evaluation, and refinement of robust algorithms and models. Its primary goal is to enable the research community to make significant strides in the analysis of lemongrass leaf conditions, offering potential benefits for both agricultural practices and disease prevention strategies.

The primary goal of this dataset's curation is to simplify the creation, evaluation, and enhancement of machine learning models and algorithms that are specifically intended to accurately identify the condition of lemongrass leaves. The dataset, which encompasses a range of conditions such as healthy, unhealthy, and dried leaves, aims to enable researchers in developing robust and effective solutions.

The dataset consists of a total of 10,042 images, distributed across categories as shown in Table 1.

**Table 1:** Distribution of lemongrass leaf images.

Sr No	Categories	Number of images
1.	Unhealthy	3822
2.	Healthy	3252
3.	Dried	2968
**Total**		**10,042**

The majority 3822 images belong to the “Unhealthy Leaves”, focusing on conditions related to plant health and potential diseases. “Healthy Leaves” contribute 3252 images, crucial for the study of plant vitality and optimal growth conditions. Additionally, 2968 images of “Dried Leaves” offer insights into leaf aging and stress conditions. This balanced distribution ensures comprehensive coverage of leaf conditions, essential for robust machine learning and data analysis in plant health and agriculture.

**Fig. 1:**
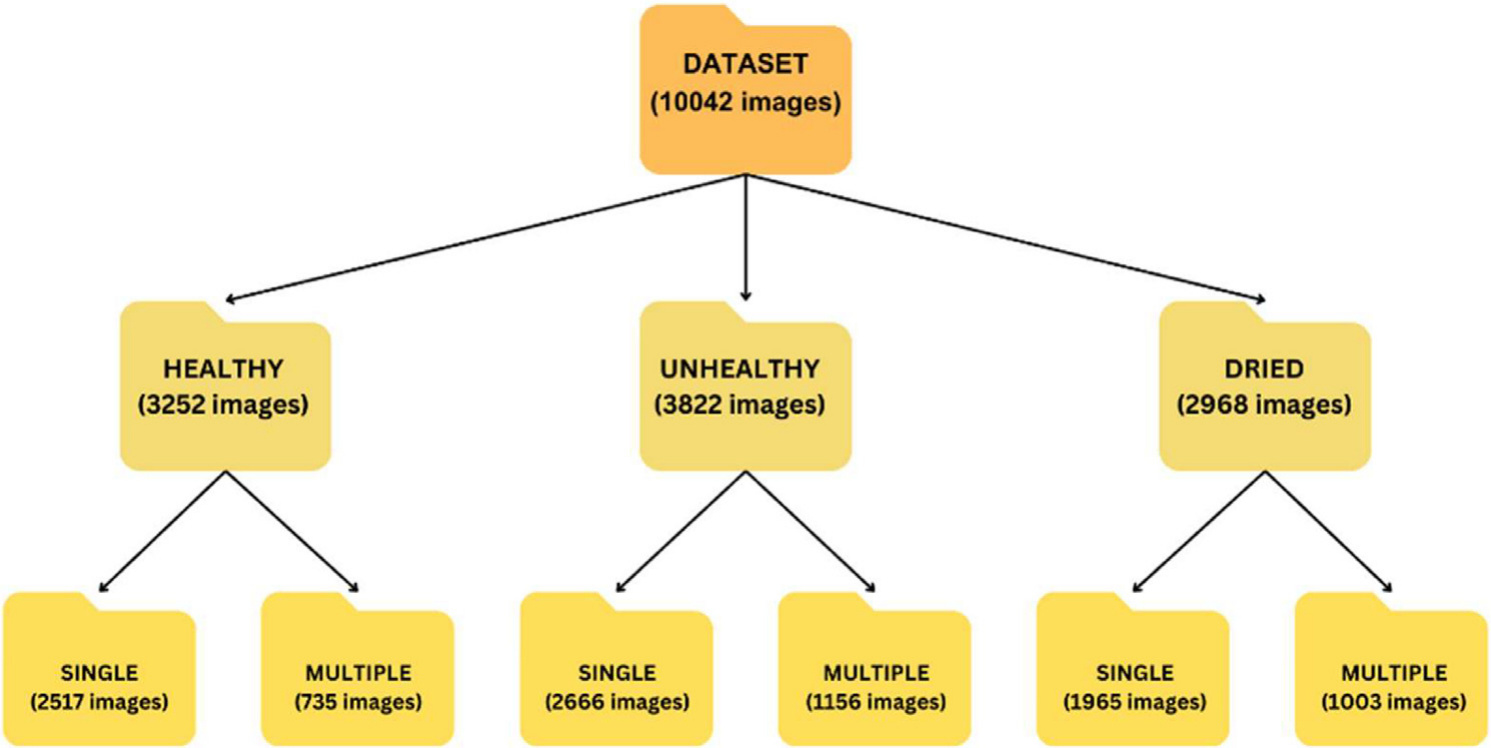
Organization of the lemongrass leaf dataset's folder structure.

The images encompass a wide range of factors, including diverse backgrounds, lighting conditions, and various camera angles, featuring single and multiple leaves. This was done on purpose to simulate real-world scenarios and challenges. Each image has been meticulously annotated to denote its specific condition category, serving as valuable ground truth labels for supervised learning tasks. These images capture leaves under varying lighting conditions, making them applicable for scenarios demanding adaptability in diverse environmental settings, where different backgrounds and various camera angles are significant considerations. Fig. 1 shows the Organization of the Lemongrass Leaf Dataset's Folder Structure.

Table 2 shows some sample images from the lemongrass leaves dataset. Each category is organized within distinct folders, ensuring straightforward access and identification of specific disease samples.

**Table 2:** Exemplar images from the lemongrass leaf dataset.

Lemongrass leaf dataset		
Category	Leaf	Leaves
**Dried**		
**Healthy**		
**Unhealthy**		

## 3 Experimental Design, Materials and Methods

### 3.1 Experimental design

**Fig. 2:**
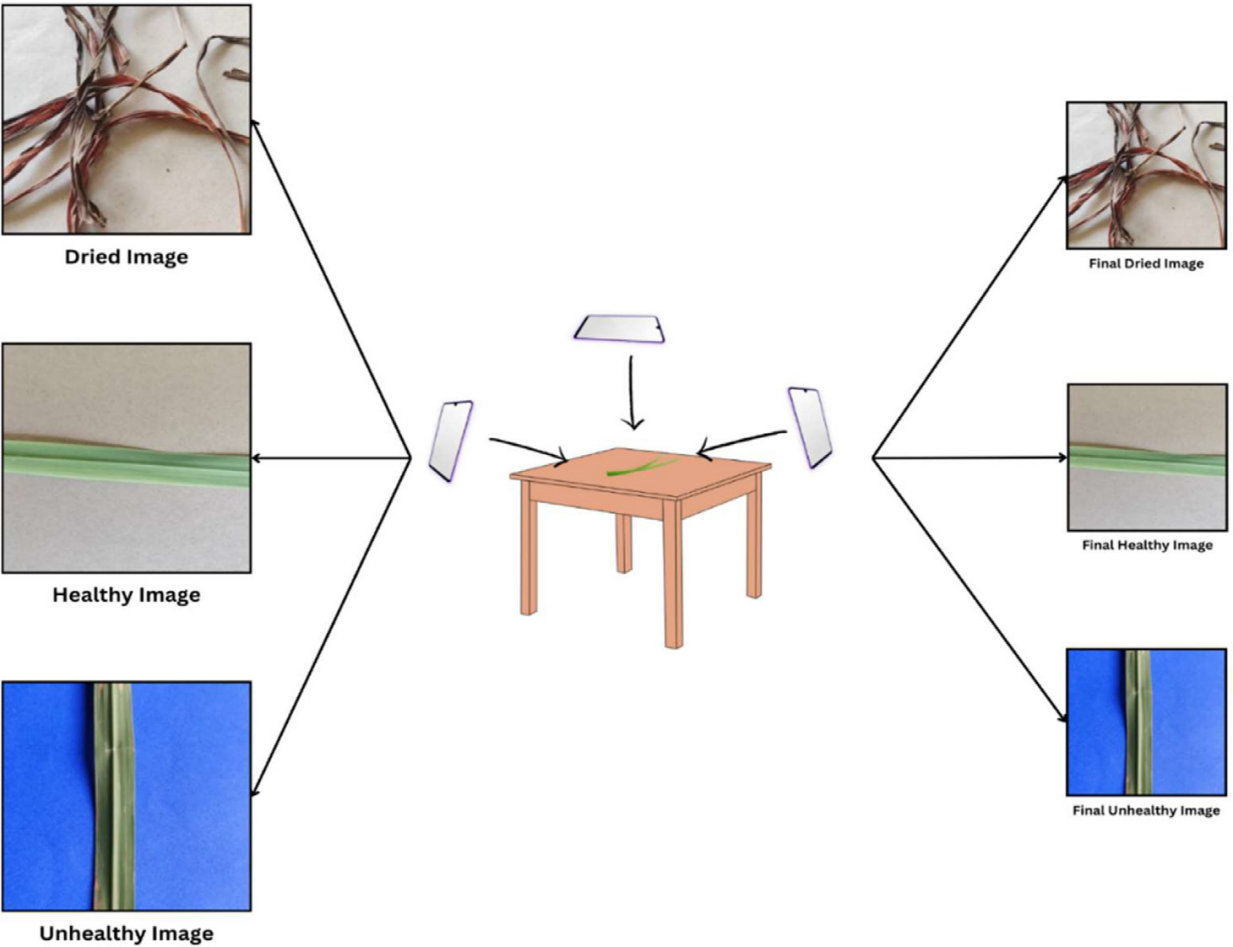
Experimental configuration for the lemongrass leaf dataset.

The experimental framework for constructing the Lemongrass leaf image dataset involved the acquisition of high-resolution images depicting Lemongrass leaves within their natural ecological context. Lemongrass specimens were systematically procured from the Vishwakarma University Pune herbal garden (18°27′34.8″N 73°53′01.1″E) over the period spanning August 2023 to October 2023. A comprehensive total of twenty individual plants were meticulously selected to facilitate leaf collection for dataset generation. Photographic documentation of all chosen plant leaves was executed across a spectrum of diverse backgrounds, angles, and lighting conditions (Fig. 2).

The images were captured from various angles and backgrounds, providing a comprehensive representation of different leaf conditions. The task of classifying the leaves was undertaken by Yogesh Suryawanshi, a PhD holder in Botany. The classification criteria were based on visual attributes, where leaves with a distinct green color and no discernible spots were categorized as “Healthy.” On the other hand, leaves showing visible spots were classified as “Unhealthy.” Dried leaves from their natural habitat were obtained, and a detailed analysis of moisture content was carried out. Leaves with a moisture content below 10% were systematically labelled as “Dried”.

**Fig. 3:**
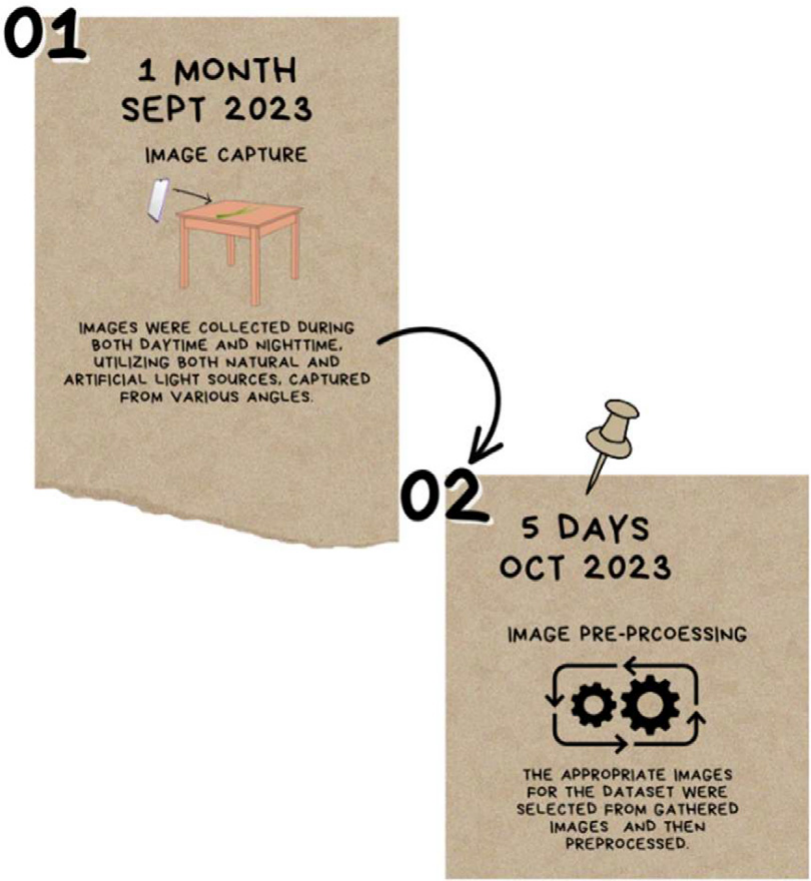
Data collection process.

**Fig. 4:**
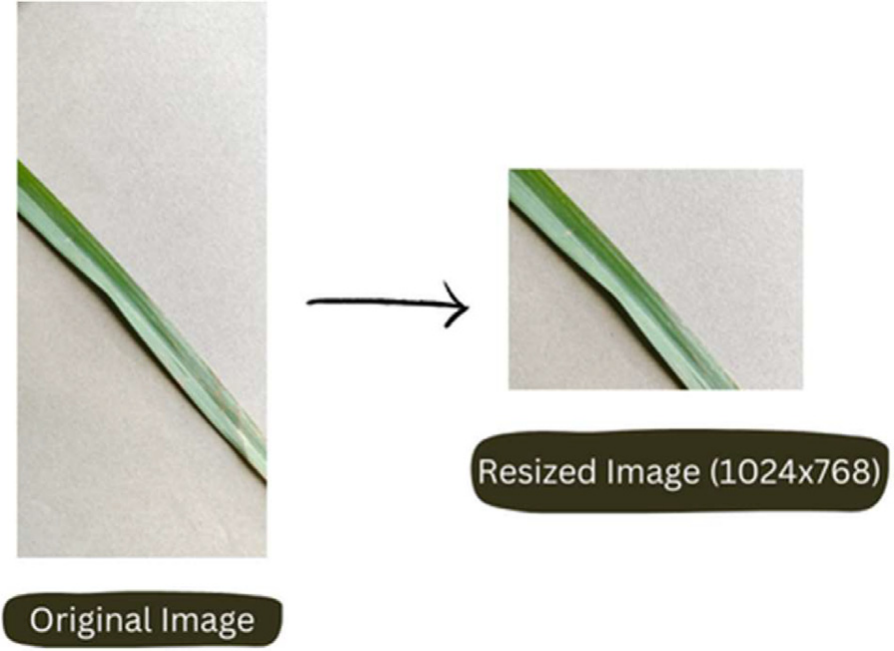
Pre-processed image.

The lemongrass leaf images were captured using the IQOO Z3 5G mobile phone, ensuring consistent image quality and resolution for each leaf sample. Fig. 2 shows the experimental setup for dataset creation. The dataset encompasses three leaf conditions (dried, healthy, and unhealthy), introducing variability in lighting and environmental factors to mimic real-world scenarios. Fig. 3 shows data acquisition process and Fig. 4 illustrates pre-processed image.

Step 1: Image Capture (September 2023) - In this step, we conducted field visits during both daytime and night time to capture images related to various lemongrass leaf conditions, including those associated with diseases. The primary objective was to compile a comprehensive collection of images relevant to lemongrass leaf conditions, especially those linked to diseases.

Step 2: Image Pre-processing (October 2023) - During this step, we enhanced the quality of lemongrass leaf images by resizing them to 1024 × 768 using FastStone Photo Resizer. The data acquisition process involved capturing images during field visits and subsequently preparing them through pre-processing for inclusion in the dataset.

### 3.2 Materials or specification of image acquisition system

The mobile phone (IQOO Z3 5G) used in the data acquisition process and the specifications of the captured images are:

Sensor Type: 64 MP GW1 / S5KGW3

Focal Length: 26 mm

Aperture Range: f/1.79-f/2.2

Aspect Ratio: 4:3

The images taken were saved in JPG format and were resized to a resolution of 1024 × 768 pixels using FastStone Photo Resizer. These specifications provide crucial details about the cameras used and the image properties obtained during the data acquisition process.

### 3.3 Pre-processing method

**Fig. 5:**
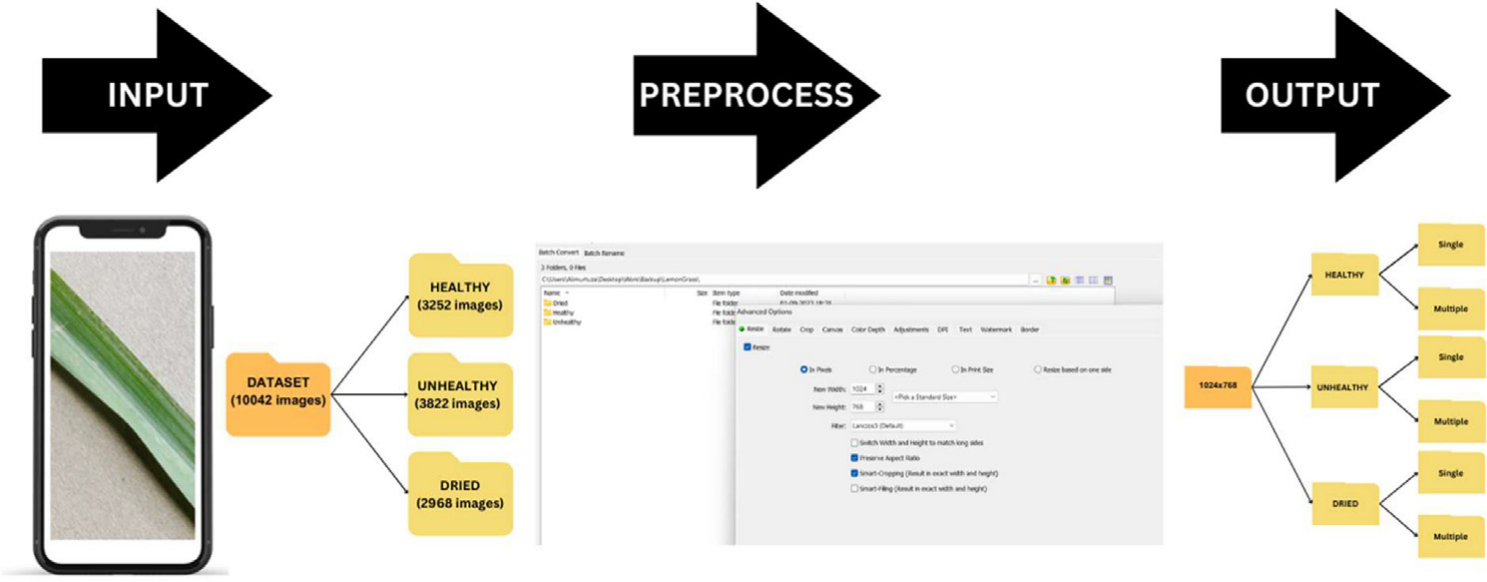
Stepwise pre-processing process.

We implemented a systematic pre-processing methodology to enhance the quality and compatibility of the dataset images. The initial step involved utilizing FastStone Photo Resizer, a versatile image processing tool renowned for its efficacy in batch image resizing. This facilitated efficient handling of large volumes of images, proving invaluable for various research applications such as image-based machine learning, image analysis, and data augmentation, as illustrated in Fig. 5.

Following the resizing process, the subsequent pre-processing step leveraged the ‘preprocess_input’ function embedded in the Keras library. Tailored for pre-trained models, this function encompasses a suite of operations, including mean subtraction, channel reordering, scaling, and resizing. These operations collectively ensure that the images are formatted in accordance with the prerequisites of the chosen pre-trained model. This meticulous pre-processing workflow not only streamlines the data for compatibility with sophisticated models but also lays the groundwork for subsequent stages of analysis, contributing to the overall robustness and efficacy of our research methodology.

### 3.4 Demonstrating the significance of the lemongrass leaves dataset

In the realm of machine learning datasets, several notable contributions have emerged recently [2], [3], [4], [5], [6], [7], [8] catering to machine learning applications. We wanted to show just how valuable our Lemongrass Leaves Dataset [1] is, so we ran some experiments using well-known pre-trained models like InceptionV3, Xception, and MobileNetV2. Our goal was to see how this dataset can boost the accuracy of machine learning models, especially when it comes to identifying Lemongrass leaves and spotting signs of disease.

First, we ran these pre-trained models without any tweaks using our dataset as a sort of benchmark. Then, we gave these models a boost by training them on our Lemongrass Leaves Dataset. What we found was pretty exciting. When we fine-tuned these models with our dataset, there was a significant jump in accuracy. This was most apparent in how well the models could detect and classify Lemongrass leaves, particularly when it came to picking up signs of disease. Table 3 shows accuracy of pretrained machine learning models on the lemongrass dataset before and after training with our dataset. Similarly, Table 4 shows confusion matrix of pretrained machine learning models on the lemongrass dataset before and after training with our dataset.

**Table 3:** Accuracy of pretrained machine learning models on the lemongrass dataset: before and after training.

Machine learning model	Accuracy (before training on our dataset)	Accuracy (after training on our dataset)
InceptionV3	34.10%	89.50%
Xception	35.54%	94.67%
MobileNetV2	38.97%	96.76%

**Table 4:** Confusion Matrix of pre-trained models.

NO	Model	Confusion Matrix (Before Training on our dataset)	Confusion Matrix (After Training on our dataset)
**1**	**InceptionV3**		
**2**	**Xception**		
**3**	**MobileNetV2**		

In a nutshell, our Lemongrass Leaves Dataset plays a crucial role in making these machine learning models, like InceptionV3, Xception, and MobileNetV2, perform much better. By offering a solid resource for training and fine-tuning, our dataset becomes a vital tool in creating more reliable models that can help improve Lemongrass cultivation and keep those plants healthy.

The above confusion matrix in the Table 4, in the context of our three-class classification problem encompassing ‘dried,’ ‘healthy,’ and ‘unhealthy’ categories, offers a detailed breakdown of the model's predictive accuracy. It allows us to discern where the model excels, correctly identifying instances within each class (true positives), and where it stumbles, making classification errors (predicted positives and predicted negatives). This nuanced evaluation is invaluable for understanding the model's behavior and guiding improvements.

### 3.5 Dataset applications and future possibilities

Practical applications of the Lemongrass Leaf Image dataset extend across diverse domains. In agriculture research, this dataset facilitates the training of machine learning models for precise leaf condition classification. Researchers can leverage its authenticity and extensive size to gain insights into Lemongrass cultivation, enhancing crop yield, and preventing diseases. For farmers, the dataset serves as a vital tool for real-time monitoring of crop health, enabling proactive disease prevention measures. Beyond agriculture, the dataset finds utility in machine learning applications, contributing to advancements in image classification algorithms. Future possibilities for the dataset include its integration into smart farming systems, enabling automated crop health assessments and providing a foundation for the development of innovative solutions in sustainable agriculture and plant health management.

## Limitations

The Lemongrass Leaf Image dataset lacks specific disease categorization.

## Ethics Statement

Our study does not involve studies with animals or humans. Therefore, we confirm that our research strictly adheres to the guidelines for authors provided by Data in Brief terms of ethical considerations.

## CRediT authorship contribution statement

**Kailas Patil:** Conceptualization, Supervision, Writing – review & editing. **Yogesh Suryawanshi:** Conceptualization, Data curation, Writing – review & editing. **Alimurtuza Patrawala:** Methodology. **Prawit Chumchu:** Writing – review & editing.

## Data Availability

Lemongrass Leaf Image Dataset: Mobile-Photographed Image Compilation (Original data) (Mendeley Data).
